# Isoform-Specific Effects of Apolipoprotein E on Hydrogen Peroxide-Induced Apoptosis in Human Induced Pluripotent Stem Cell (iPSC)-Derived Cortical Neurons

**DOI:** 10.3390/ijms222111582

**Published:** 2021-10-27

**Authors:** Huiling Gao, Wei Zheng, Cheng Li, He Xu

**Affiliations:** 1 College of Life and Health Sciences, Northeastern University, Shenyang 110819, China; gaohuiling@mail.neu.edu.cn; 2 Department of Histology and Embryology, China Medical University, Shenyang 110122, China; wzheng@cmu.edu.cn; 3 Department of Immunology, China Medical University, Shenyang 110122, China; licheng@cmu.edu.cn; 4 Department of Histology and Embryology, Faculty of Medicine, Shenzhen University, Shenzhen 518061, China

**Keywords:** apolipoprotein E, apoptosis, iPSC-derived cortical neurons, Alzheimer’s disease, neurodegeneration

## Abstract

Hydrogen peroxide (H_2_O_2_)-induced neuronal apoptosis is critical to the pathology of Alzheimer’s disease (AD) as well as other neurodegenerative diseases. The neuroprotective effects of apolipoprotein (ApoE) isoforms against apoptosis and the underlying mechanism remains controversial. Here, we have generated human cortical neurons from iPSCs and induced apoptosis with H_2_O_2_. We show that ApoE2 and ApoE3 pretreatments significantly attenuate neuronal apoptosis, whereas ApoE4 has no neuroprotective effect and higher concentrations of ApoE4 even display toxic effect. We further identify that ApoE2 and ApoE3 regulate Akt/FoxO3a/Bim signaling pathway in the presence of H_2_O_2_. We propose that ApoE alleviates H_2_O_2_-induced apoptosis in human iPSC-derived neuronal culture in an isoform specific manner. Our results provide an alternative mechanistic explanation on how ApoE isoforms influence the risk of AD onset as well as a promising therapeutic target for diseases involving neuronal apoptosis in the central nervous system.

## 1. Introduction

The apolipoprotein E gene (*APOE*) has three major variants, *APOE2*, *APOE3* and *APOE4*; so far *APOE* has been confirmed as the strongest genetic risk modifier for late-onset Alzheimer’s disease (AD), with the *APOE4* conferring an increased risk and the *APOE2* conferring a decreased risk relative to the common *APOE3* allele [1,2]. Brain apolipoprotein E (ApoE) is a cholesterol transport protein secreted primarily by astrocytes that plays a vital role in transporting cholesterol and other lipids to neurons through ApoE receptors, a group of endocytic receptors belonging to low-density lipoprotein receptor (LDLR) family [3]. Compelling evidence has demonstrated the disparate effects of ApoE isoforms on AD risk level is mainly attributed to ApoE isoform-specific effects on the formation of amyloid plaques and neurofibrillary tangles (NFTs) [4]. 

Cell apoptosis indeed plays critical roles in neuronal loss in neurodegenerative diseases [5,6]. One of the important stimuli for apoptosis is the increased oxidative stress caused by excessive formation of hydrogen peroxide and hydroxyl radicals in the brain [7,8]. There’s evidence showing that oxidative imbalance is a manifestation of AD that even precedes the formation of Aβ plaques and NFTs [9]. Execution of apoptosis is ensured by the activation of cysteine proteases cascades (caspases). Caspase 3 is the final apoptosis executioner caspase that can be activated by various apoptotic pathways. Furthermore, caspase 3 activity has been found to be elevated in the brain of neurodegenerative disease patients [6,10]. Forkhead Box O3a (FoxO3a), a member of class O of forkhead box transcription factors, has been implicated in neuronal apoptosis via regulating its downstream target genes [11]. Multiple post-translational modifications, especially phosphorylation, serve a major role in FoxO3a regulation [12]. Three major FoxO3a phosphorylation sites regulated by Akt (also called protein kinase B) have been identified, including threonine32 (Thr32), serine253 (Ser253) and serine315 (Ser315) [13]. When phosphorylated by Akt, FoxO3a is associated with 14-3-3 protein in the cytosol and excluded out from the nucleus to promote cell survival, whereas reducing Akt activity results in the nuclear translocation of FoxO3a and drive multiple downstream genes expression, such as *BIM*, *FASL* (Fas ligand) and *PUMA* (p53 upregulated modulator of apoptosis), triggering apoptosis [13,14]. Bim, a pro-apoptotic protein, is required for Aβ-induced neuronal apoptosis. In addition, previous studies have shown its level is markedly elevated in AD brains [15,16]. On the other hand, c-Jun N-terminal Kinase (JNK), a member of the oxidative stress-activated mitogen-activated protein kinase family, also regulates FoxO3a activity by phosphorylation. FoxO3a has been demonstrated to be phosphorylated by JNK at the site of serine574 (Ser574) under oxidative stress condition and then accumulate in the nucleus to promote downstream pro-apoptotic gene expressions, leading to cell death [17].

The focus of current research on AD is shifting from the traditional Aβ/tau-dependent mechanism to alternative or innovative areas, such as ApoE, oxidative stress, cell aging/death mechanisms, neuroinflammation and brain metabolism [18,19]. Furthermore, H_2_O_2_-induced neuronal apoptosis has been detected not only in AD but also in other neurodegenerative diseases [20]. In the present study, we established the cell model of human iPSC-derived cortical neurons and generated ApoE proteins in HEK293 system to investigate the role of ApoE in neuronal apoptosis. Our findings demonstrate that ApoE protects human iPSC-derived cortical neurons from H_2_O_2_-induced apoptosis via regulating Akt/FoxO3a/Bim signaling pathway in an isoform-specific manner, with ApoE2 and ApoE3, but not ApoE4 showing neuroprotective effects. 

## 2. Results

### 2.1. Generation and Characterization of Human iPSC-Derived Cortical Neurons

In our study, four iPSC lines carrying *APOE3/E3* were utilized (see Table 1, YK26 and YK27 are two iPSC clones generated from one person). All iPSCs displayed typical characteristics of stem cells, including positive immunostaining of the pluripotency-associated markers: OCT4, SSEA4 and TRA-1-81 (Figure S1a); gene expressions of endogenous *OCT4*, *NANOG* and *LIN28*, comparable with human H9-ESC line (WiCell Research Institute, Madison, WI, USA) (Figure S1b); and ability of embryoid body (EB) formation and differentiation into ectoderm (*TUJ1*), mesoderm (*SMA*) and endoderm (*AFP*) (Figure S1c). 

There is no qualitative difference between four iPSC lines in cell morphology, proliferation, and the ability to remain undifferentiated in culture. All iPSC lines in our study are from people carrying *APOE3/E3* genotype, so that we can exclude the confounding influence of different endogenous ApoE isoforms. 

**Figure 1:**
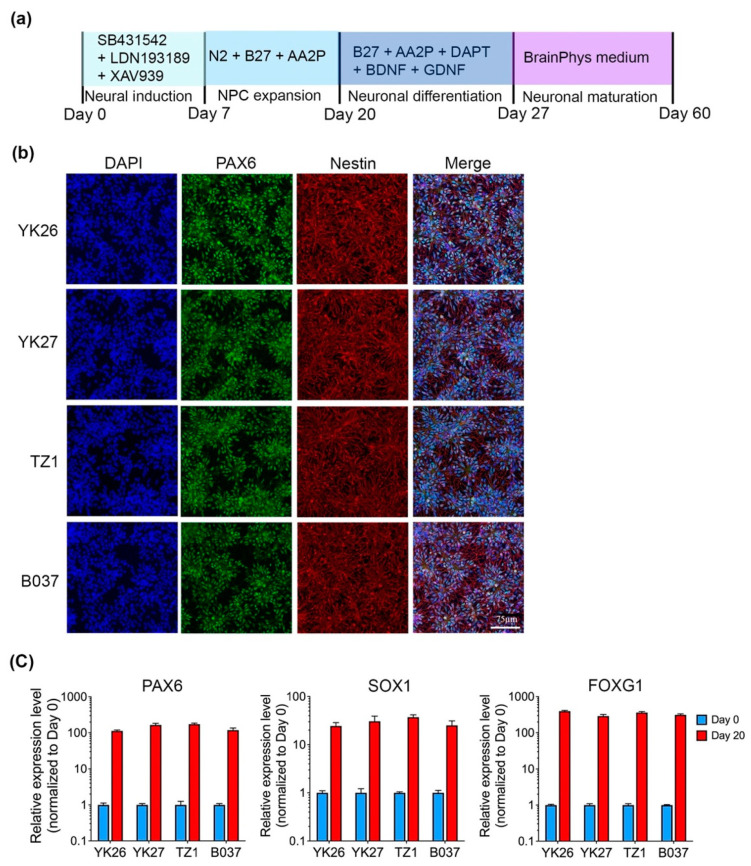
Differentiation of iPSCs into Neural progenitor cells (NPCs). (**a**) Scheme of neuronal differentiation protocol. (**b**) Cells derived from four iPSC lines are confirmed as NPCs on day 20, showing immunopositive staining for neural stem cell makers: Nestin and PAX6. White scale bar, 75 μm. (**c**) *PAX6*, *SOX1*, and *FOXG1* genes are highly expressed in NPCs as assessed by qRT-PCR. *n* = 4 experiments for each line were repeated independently, data are presented as mean ± standard error of mean (SEM).

We employed an established SMAD inhibition method with some modifications to generate forebrain cortical neurons with functional electrophysiological properties by day 60 of differentiation, in the absence of glial cell co-culture (Figure 1a). After 20 days of neural induction, iPSCs were differentiated to neural progenitor cells (NPCs) robustly expressing PAX6 and Nestin (Figure 1b). qRT-PCR results showed that NPC markers (*PAX6* and *SOX1*) and forebrain marker (*FOXG1*) levels were significantly elevated to more than 100 folds on day 20 compared with day 0 (Figure 1c). 

**Figure 2:**
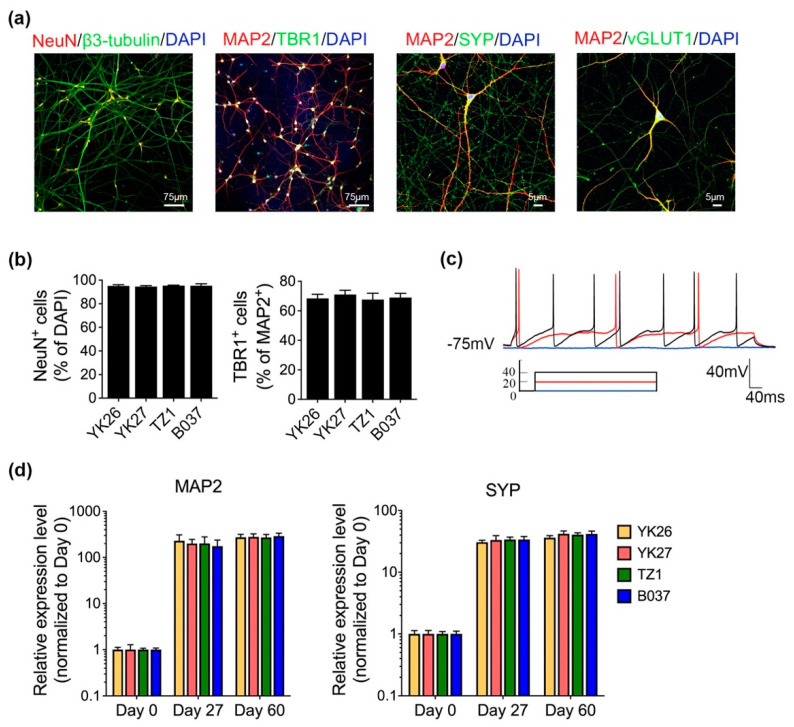
Characterization of mature cortical neurons. (**a**) Representative confocal images show neuronal markers expression on day 60: MAP2, β3-tubulin, NeuN and TBR1. White scale bar, 75 μm; synaptic markers expressed includes: synaptophysin (SYP) and vesicular glutamate transporter 1(vGLUT1). White scale bar, 5 μm. (**b**) More than 95% cells are NeuN+ and around 70% cells are TBR1+ in the neuronal culture. At least five random fields were counted per coverslip. (**c**) Actional potential exists in day 60 neurons. *n* = 8–10 neurons were tested per coverslip. (**d**) Elevated mRNA levels of *MAP2* and *SYP* over induction period. *n* = 4, data are presented as mean ± SEM.

NPCs were subsequently differentiated into glutamatergic neurons (vGLUT1+) expressing neuronal markers NeuN, β3-tubulin and MAP2 at day 60 (Figure 2a). More than 95% of total cells were NeuN+ and approximately 70% of cells were TBR1+ (layer VI of human cortex) as measured by immunofluorescence (IF) (Figure 2b), indicating an efficient differentiation of cortical neurons. In addition, presynaptic marker, synaptophysin (SYP), was also detected (Figure 2a), suggesting the formation of synapses in the culture. Furthermore, action potentials (APs) can be evoked (Figure 2c), implying neurons were functional on day 60. A time-course analysis showed that both *MAP2* and *SYP* mRNA expression levels were highly elevated on day 27 and day 60 of differentiation (Figure 2d).

### 2.2. ApoE2 and ApoE3 Prevent Neurons from H_2_O_2_-Induced Apoptosis via Binding to ApoE Receptor(s)

**Figure 3:**
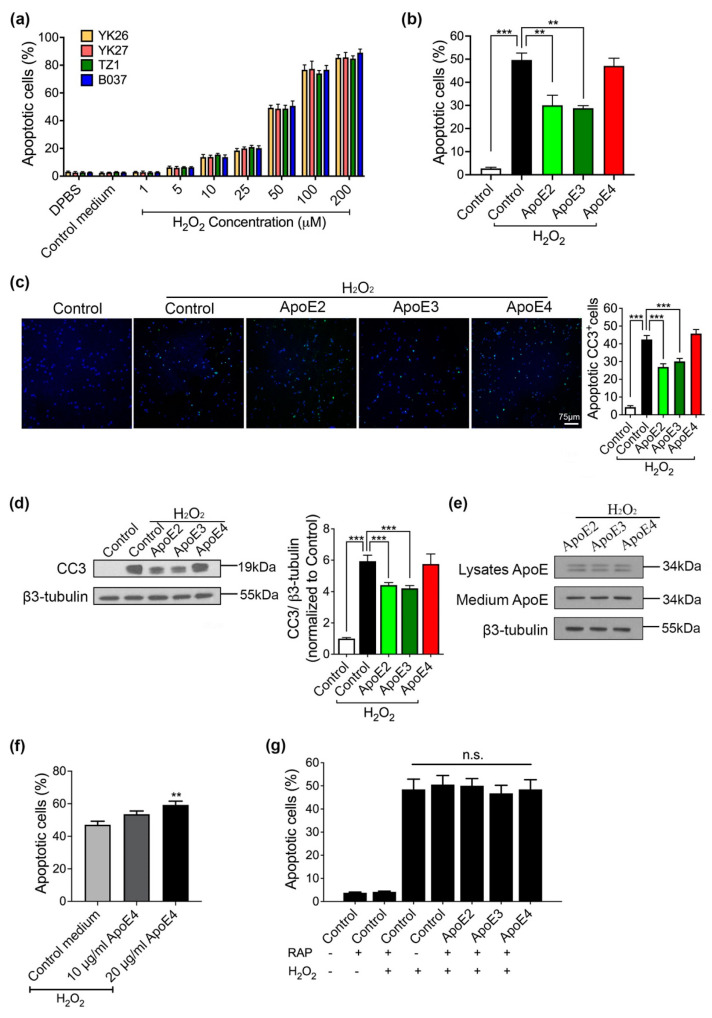
ApoE2 and ApoE3 prevent neurons from H_2_O_2_-induced apoptosis through ApoE receptor. (**a**) 6 h treatment of H_2_O_2_ caused neuronal apoptosis in a dose-dependent manner. (**b**) Apoptosis assay for neurons treated with control medium or neurons co-treated with ApoE isoforms and H_2_O_2_. The percentage of apoptosis was evaluated by HT TiterTACS Apoptosis Detection Kit. (**c**) Confocal images showing cleaved caspase 3 (CC3) positive cells under different conditions and quantification was performed. At least five random fields were counted per coverslip. White scale bar, 75 µm. (**d**) Western blot analysis comparing CC3 level between control medium and ApoE isoforms treated neurons in the absence or presence of H_2_O_2_. (**e**) Western analysis of both intracellular and extracellular ApoE levels after H_2_O_2_ treatment. (**f**) Apoptosis level was measured with neurons co-treated with ApoE4 (10 and 20 µg/mL) and H_2_O_2_. (**g**) Day 60 neurons were pretreated with 200 nM RAP for 2 h before adding ApoE-containing or control medium. Apoptosis assay was performed. *n* = 4 per line, data are presented as a summary of all lines (mean ± SEM) using one-way ANOVA and Dunnett test. ** *p* < 0.01, *** *p* < 0.001 versus neurons co-treated with control medium and H_2_O_2_. n.s.: non-significant differences.

We observed that the apoptosis level in control medium (harvested from control- (FUGW-) transfected 293-F cells) treated neurons is similar to neurons treated with DPBS. Very few neurons (<5%) underwent apoptosis without H_2_O_2_ treatment, suggesting that our neuronal culture was healthy. With H_2_O_2_ treatment (1–200 µM, 6 h), we found that apoptosis level increased in a dose-dependent manner with no significant difference between lines (Figure 3a). In the following experiments, we treated neurons with 50 μM of H_2_O_2_ which caused approximately 50% (49.3 ± 4.23%) of total cells to become apoptotic.

ApoE isoforms were prepared and measured following an established protocol [21]. The concentrations of HEK293-derived ApoE in the harvested medium are typically 400–600 µg/mL (80–120×) (Figure S2a). Western blot results showed that day-60 neurons derived from all human iPSC lines produced similar amount of ApoE intracellularly (Figure S2b), while no ApoE was detected in the medium. Media containing different ApoE isoforms were added to day-60 neuronal cultures for 12 h (ApoE final concentration was 5 μg/mL), and we did not observe any toxic effect on cell viability (Figure S2c). 

The neuroprotective effect of ApoE isoforms was evaluated with an apoptosis assay. Day-60 neurons were pretreated with ApoE medium (5 µg/mL) or control medium (medium without ApoE) for 6 h and then stressed with 50 µM H_2_O_2_ for another 6 h in the same medium. Treatment of control medium plus H_2_O_2_ caused the apoptotic rate up to 49.6% (±2.97%). ApoE2 and ApoE3 pretreatment achieved protective effects, with apoptosis percentage reducing to 30.1% (±4.33%) and 28.8 % (±1.90%), respectively (Figure 3b). However, ApoE4 pretreatment had no protective effect on H_2_O_2_-induced apoptosis with 47.1% (±5.75%) apoptotic neurons (Figure 3b). These findings were confirmed by the analysis of a critical cell apoptosis indicator, caspase 3. Notably, 42.25% (±2.24%) cells expressed the cleaved caspase 3 (CC3) after H_2_O_2_ treatment, while 26.9% (±1.82%) and 31.1% (±1.66%) neurons were positive for CC3 in the group of ApoE2 and ApoE3 (Figure 3c). Western blot results showed that caspase activity (indicated by CC3 level) was significantly elevated by H_2_O_2_, which could be alleviated by ApoE2 and ApoE3, indicative of neuroprotective function (Figure 3d). It has been demonstrated that ApoE4 is much more susceptible to proteolysis than ApoE2 and ApoE3 [22], and in the presence of H_2_O_2_ ApoE4 might be more prone to degradation which in turn may explain why it failed to protect neurons from apoptosis. Therefore, we investigated both the intracellular and extracellular ApoE levels of neurons pretreated with different ApoE isoforms. We observed no difference in ApoE levels in neuronal lysates or medium among ApoE-treated groups (Figure 3e), indicating that the isoform-specific anti-apoptotic effect of ApoE was not due to different amount of intracellular or extracellular ApoE isoforms. Furthermore, we tested whether higher amount of ApoE4 treatment (10 and 20 µg/mL) could be neuroprotective in the presence of H_2_O_2_. Neurons pretreated with 20 µg/mL of ApoE4 underwent more apoptosis (59.26 ± 2.3%) compared with neurons pretreated with control medium (47.01 ± 2.18%) (Figure 3f), indicative of a toxic effect. These results demonstrated that ApoE2 and ApoE3, but not ApoE4, could protect human cortical neurons against H_2_O_2_-induced apoptosis.

In addition, day-60 neurons were pretreated with RAP (200 nM) for 2 h before ApoE-containing medium or control medium was applied. Receptor associated protein (RAP) can compete with ligands for binding to LDLR family [23], and itself does not induce apoptosis in neurons. 

We found that RAP treatment abrogated the protective effects of ApoE2 and ApoE3, resulting in 50.07% (±3.21%) and 46.77% (±3.78%) apoptotic cells, respectively (Figure 3g). These results implied that the neuroprotection of ApoE2 and ApoE3 against H_2_O_2_-induced apoptosis is mediated by LDLR family members.

### 2.3. ApoE2 and ApoE3 Regulate Akt/FoxO3a Signaling Pathway in the Presence of H_2_O_2_

**Figure 4:**
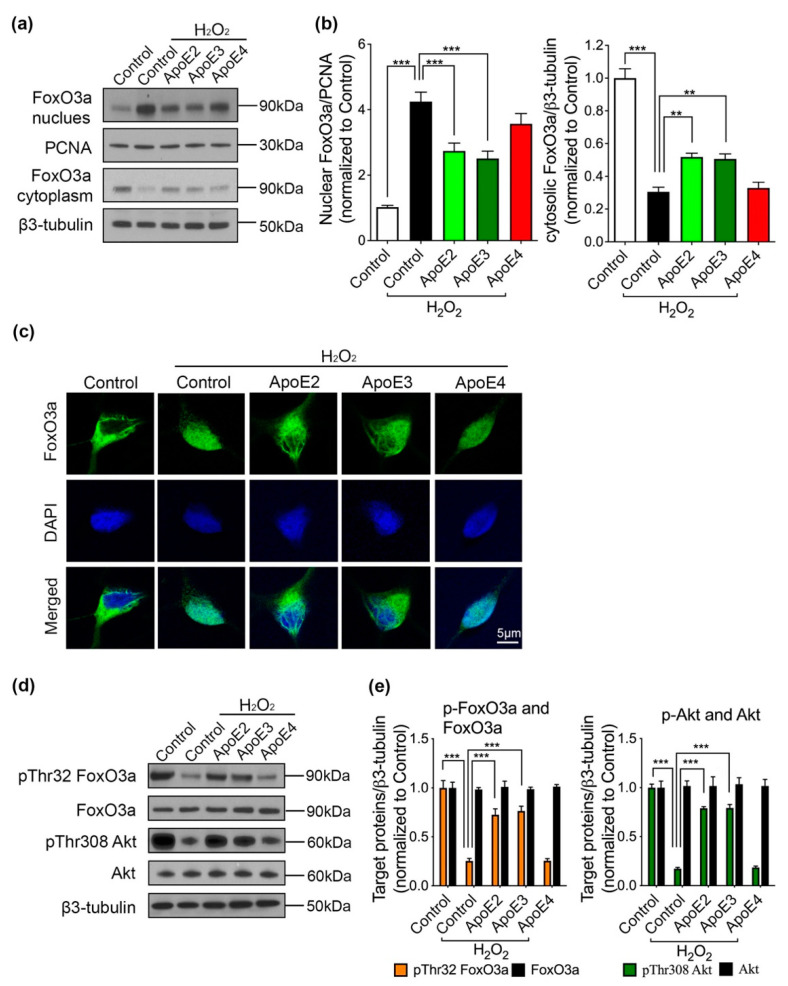
ApoE2 and ApoE3 regulate Akt/FoxO3a signaling pathway in response to H_2_O_2_. (**a**,**b**) Both nuclear and cytosolic FoxO3a protein expression levels were measured by Western blot and quantitative analysis was performed. Equal loading and protein transference were checked by detection of β3-tubulin and proliferating cell nuclear antigen (PCNA) for cytosolic and nuclear extracts, respectively. (**c**) Representative immunofluorescence images exhibiting the translocation of FoxO3a in neurons under different conditions. White scale bar, 5 μm. (**d**,**e**) Expression levels of phosphorylated FoxO3a (pThr32) and Akt (pThr308) were detected by Western blot with specific antibodies and quantitative analysis was performed. Total FoxO3a and Akt were also measured as reference. *n* = 4 per line, data are presented as a summary of all lines (mean ± SEM) using one-way ANOVA and Dunnett test. ** *p* < 0.01, *** *p* < 0.001 versus neurons co-treated with control medium and H_2_O_2_.

Following the above findings that ApoE2 and ApoE3 prevented neurons from apoptosis, we aimed to elucidate the underlying mechanism. We found that H_2_O_2_ increased nuclear FoxO3a level to more than 4 folds and decreased cytosolic FoxO3a level to around one third of those in neurons without H_2_O_2_ treatment, indicating that FoxO3a translocated to the nucleus in response to H_2_O_2_ treatment (Figure 4a,b). Strikingly, ApoE2 and ApoE3 treatment could reduce H_2_O_2_-induced FoxO3a translocation to some extent, whereas ApoE4 pretreatment had no effect (Figure 4a,b). In addition, FoxO3a immunostaining images showed that FoxO3a (green) was present mostly in the cytosol of cortical neurons derived from iPSCs in the absence of H_2_O_2_, while a large amount of FoxO3a colocalized with the nuclear marker DAPI (blue) after 6 h H_2_O_2_ treatment (Figure 4c). In comparison with neurons co-treated with control medium and H_2_O_2_, less nuclear colocalization of FoxO3a and DAPI was detected in the neurons pretreated ApoE2 or ApoE3, but not ApoE4 (Figure 4c), suggesting that ApoE2 and ApoE3 regulated FoxO3a subcellular location in response to H_2_O_2_. Moreover, we found ApoE isoforms themselves did not affect FoxO3a subcellular location (Figure S3a,b).

FoxO3a phosphorylation has been demonstrated to regulate FoxO3a transcriptional activity via a cytoplasmic-nuclear shuttle mechanism [11]. In the present study, pThr32 FoxO3a level was significantly decreased by H_2_O_2_. More importantly, pretreatment of ApoE2 and ApoE3, but not ApoE4, could attenuate H_2_O_2_-induced decrease in pThr32 FoxO3a level (Figure 4d,e). 

These alterations in pThr32 FoxO3a level was consistent with our previous observations in FoxO3a translocation. Furthermore, the level of phosphorylated Akt at the site of 308 (pThr308 Akt) was also remarkably reduced by H_2_O_2_ treatment, suggesting that Akt activity was inhibited by H_2_O_2_. Pretreating ApoE2 or ApoE3 caused an obvious recovery of Akt activity, showing an obvious increase in pThr308 Akt level compared with control medium pretreatment in the presence of H_2_O_2_. Meanwhile, ApoE4 pretreatment did not affect Akt activity (Figure 4d,e). These data indicated that Akt/FoxO3a signaling pathway was regulated by ApoE2 and ApoE3 to prevent H_2_O_2_-induced neuronal apoptosis.

Additionally, JNK phosphorylates FoxO3a at the site of Ser574 upon activation, resulting in nuclear accumulation of FoxO3a and enhanced FoxO3a activity, which antagonizes Akt signaling pathway [17,24]. We found that both p-JNK 1/2 level (indicating JNK activity) and pSer574 FoxO3a level were upregulated by H_2_O_2_ (Figure S4a,b). ApoE isoforms pretreatment did not change their levels compared with control medium pretreatment, implying that the JNK/FoxO3a signaling pathway did not contribute to the anti-apoptotic effects of ApoE. A previous study has shown that ApoE protects retinal ganglion cells from apoptosis in an order of ApoE3 > ApoE4 by inactivating GSK3β, a proapoptotic kinase that is inactivated by phosphorylation at Serine 9 (pSer9 GSK3β) [23]. Although pSer9 GSK3β level was decreased by H_2_O_2_, there’s no significant difference in pSer9 GSK3β level between ApoE-treated and control medium-treated neurons in the presence of H_2_O_2_ (Figure S4c,d), suggesting that GSK3β may contribute to the H_2_O_2_-induced apoptosis in our neuronal culture, but was not involved in the anti-apoptotic effects of ApoE2 and ApoE3.

### 2.4. PI3K Mediates ApoE2 and ApoE3 Neuroprotection against Apoptosis

**Figure 5:**
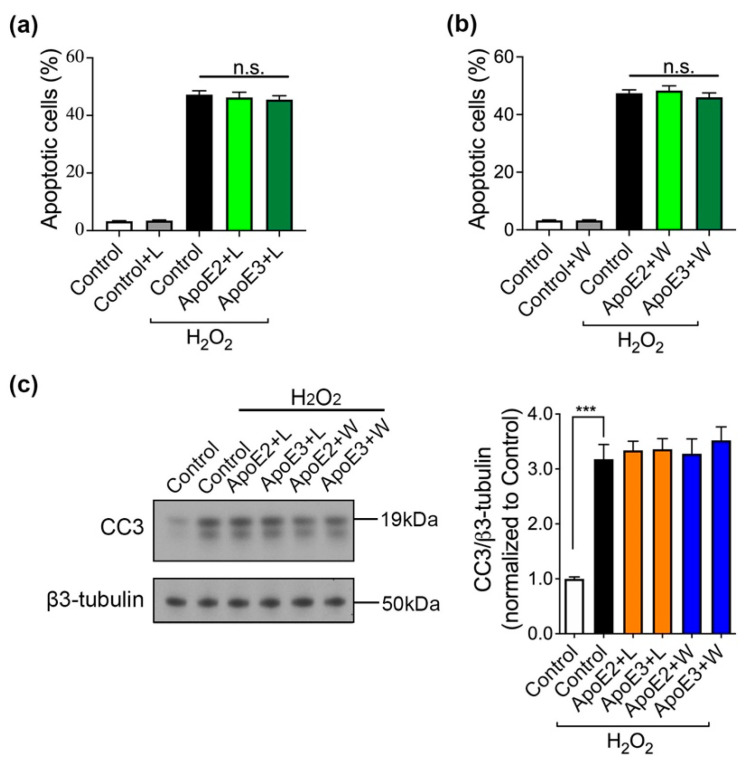
PI3K inhibitors abrogate the neuroprotective effects of ApoE2 and ApoE3. (**a**) Cell apoptosis was measured in the presence or absence of 10 μM LY294002. (**b**) Cell apoptosis was measured in the presence or absence of 50 nM wortmannin. (**c**) The alterations in the expression levels CC3 were examined by Western blot; quantification of protein expression levels and normalized to the Control group. Values are means ± SEM of four independent experiments of each line. One-way ANOVA and Dunnett test used, *** *p* < 0.001 versus neurons co-treated with control medium and H_2_O_2_. n.s.: non-significant differences.

PI3K/Akt signaling pathway is closely associated to cellular survival in various cell types [25], so we tested the efficacy of two specific inhibitors of PI3K (wortmannin and LY294002) in ApoE neuroprotection. Day-60 neurons were pretreated with 10 μM LY294002 (L) or 50 nM wortmannin (W) for 2 h before incubation with ApoE or control medium. Apoptosis assay data showed that W and L themselves were neutral to neurons, whereas W and L pretreatments abolished the protective effects of ApoE2 and ApoE3, resulting in around 50% apoptotic cells in the culture (Figure 5a,b). Moreover, Western blot results showed that ApoE2 and ApoE3 did not attenuate H_2_O_2_-induced increase in CC3 levels when neurons were pretreated with W or L (Figure 5c), suggesting that PI3K mediated the anti-apoptotic effect of ApoE2 and ApoE3.

### 2.5. Bim, A Downstream Pro-Apoptotic Effector of FoxO3a, Is Regulated by ApoE2 and ApoE3

**Figure 6:**
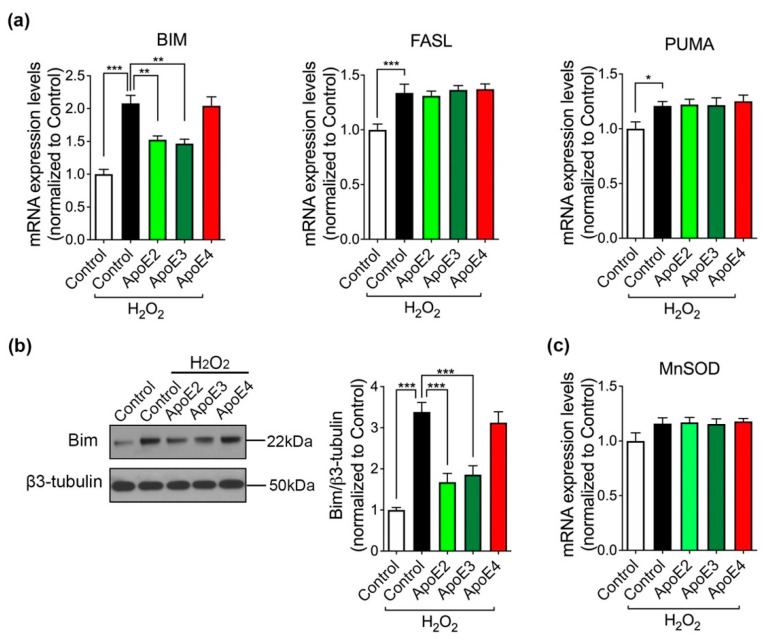
Investigations of FoxO3a downstream targets. (**a**) qRT-PCR of FoxO3a downstream pro-apoptotic targets, including *BIM*, *FASL*, *PUMA*. (**b**) Western blot analysis of Bim protein alterations caused by H_2_O_2_ and ApoE. (**c**) mRNA level of *MnSOD* was assessed by qRT-PCR. *n* = 4 per line, data are presented as a summary of all lines (mean ± SEM) using one-way ANOVA and Dunnett test. * *p* < 0.05, ** *p* < 0.01, *** *p* < 0.001 versus neurons co-treated with control medium.

We next conducted a series of experiments to define which downstream gene targets of FoxO3a was involved. As shown in Figure 6a, the mRNA levels of *BIM*, *FASL* and *PUMA* were significantly increased in response to H_2_O_2_. *BIM* mRNA level was doubled by H_2_O_2_ treatment compared with control medium treatment only. Notably, ApoE2 and ApoE3 pretreatments alleviated the H_2_O_2_-induced increase in *BIM* mRNA level, but not *FASL* or *PUMA* mRNA levels. Accordingly, pretreating ApoE2 and ApoE3 also alleviated H_2_O_2_-induced increase in Bim protein level (Figure 6b), indicating that ApoE2 and ApoE3 may rescue neurons through FoxO3a/Bim signaling pathway. On the other hand, manganese superoxide dismutase (*MnSOD*) is another FoxO3a downstream target gene and it promotes cell survival by exerting antioxidant effects [26,27]. We observed that *MnSOD* gene expression level showed a non-significant increasing trend after H_2_O_2_ treatment, which could be a protective reaction. However, its level was not regulated by ApoE isoforms treatment in the presence of H_2_O_2_ (Figure 6c), suggesting that *MnSOD* was not involved in the neuroprotection of ApoE2 and ApoE3.

## 3. Discussion

Neuronal apoptosis is a characteristic feature of many neurodegenerative disorders, such as AD [5]. Hydrogen peroxide production is promoted during neurodegeneration, which is a critical cause of brain cell apoptosis [28]. Here, we employed cortical neurons differentiated from human iPSC to evaluate the effect of ApoE isoforms on neuronal apoptosis and have identified the differential efficacies of ApoE2, ApoE3, and ApoE4 in neuroprotection: ApoE2 and ApoE3, but not ApoE4, attenuate H_2_O_2_-induced neuronal apoptosis through regulating Akt/FoxO3a/Bim signaling pathway.

CNS ApoE is produced primarily by astrocytes. In addition, microglia can greatly upregulate ApoE expression upon activation and neurons also produce ApoE under certain conditions [29]. Brain ApoE levels measured by ELISA and Western blot are highest in *APOE2* carriers and lowest in *APOE4* carriers [30]. ApoE is also synthesized by other tissues, including the liver, adipose tissue, the kidney, and the adrenal glands [31]. ApoE expression is most abundant in the liver and hepatocytes is the major source of plasma ApoE [32]. Human plasma ApoE levels follow an order of *APOE2* > *APOE3* > *APOE4* [33]. In *APOE3/E4* individuals, the amount of ApoE4 is greater than ApoE3 in the CNS but less ApoE4 than ApoE3 is found in the plasma, suggesting that the metabolic pathways of ApoE isoforms differ between the CNS and plasma [34,35].

A recent study demonstrated that neuronal apoptosis as well as synaptic loss are much more severe in cerebral organoids derived from AD patients carrying *APOE4* than those carrying *APOE3* [36]. Endogenous ApoE4 of iPSC-derived neurons predisposes neurons to cell death, while endogenous ApoE3 does not [37]. From the above, some interactive mechanism between neuronal apoptosis/death and ApoE toward neurodegeneration may exist. Our study revealed that pretreatment of ApoE2 or ApoE3 attenuated H_2_O_2_-induced increase in apoptotic cell percentage in the human iPSC-derived neuronal culture. Inhibitory protein RAP was utilized to block the binding ability of ApoE to LDLR family members, by which we identified ApoE receptor(s) mediated the neuroprotection of ApoE2 and ApoE3. However, same (physiological) concentration of ApoE4 showed no protective effect against neuronal apoptosis. Although it has been reported that ApoE4 is less stable than ApoE2 and ApoE3 [22], we did not observe any significant difference in intracellular or extracellular ApoE levels after H_2_O_2_ treatment between ApoE isoforms, suggesting that the isoform-specific effects of ApoE on H_2_O_2_-induced neuronal apoptosis is not likely due to the degradation of ApoE4 in the neuronal medium. Furthermore, we found that higher concentrations of ApoE4 could even exaggerate the neuronal apoptosis, which is consistent with other studies [38,39]. 

ApoE modulates multiple pathways in the brain in an isoform-specific manner, including lipid transport, synaptic integrity and plasticity, glucose metabolism and cerebrovascular function [29,40,41]. The isoform-specific anti-apoptotic effects of ApoE may be due to the different signaling pathways they regulated or different efficacies in regulating some cell survival/apoptosis-related pathway. FoxO3a has been shown to response to oxidative stress [42]. Nuclear FoxO3a is the active form of FoxO3a that upregulates multiple apoptosis-related genes, while cytoplasmic FoxO3a has no transcriptional activity [15]. We found a translocation of FoxO3a from cytoplasm to nucleus in neurons by H_2_O_2_ treatment, which could be alleviated by ApoE2 and ApoE3, but not ApoE4. Activation of FoxO3a by H_2_O_2_ is mainly controlled through inhibiting PI3K-Akt and activating JNK [26]. FoxO3a phosphorylation by Akt at Thr32 leads to acute translocation of FoxO3a out of the nucleus, promoting cell survival [13]. In the present study, the alterations in pThr32 FoxO3a level coincided with our findings on FoxO3a translocation after ApoE and H_2_O_2_ treatment. ApoE2 and ApoE3 were found to remarkably alleviate H_2_O_2_-induced decrease in both pThr32 FoxO3a and pThr308 Akt levels, suggesting that Akt/FoxO3a signaling pathway was regulated by ApoE2 and ApoE3 to protect against neuronal apoptosis. Moreover, we found that PI3K inhibitors abolished the anti-apoptotic effects of ApoE2 and ApoE3, indicating that PI3K mediated the neuroprotective effects of ApoE2 and ApoE3. The elevation of JNK phosphorylation level contributes to the cell death caused by H_2_O_2_ [43] and JNK phosphorylates FoxO3a at Ser574, leading to FoxO3a accumulating in the nucleus and downstream pro-apoptotic genes expression upregulation [11]. Although we observed that H_2_O_2_ caused an obvious increase in both p-JNK1/2 and pSer574 FoxO3a levels, pre-existing ApoE2 or ApoE3 did not alter their levels, suggesting that JNK/FoxO3a signaling pathway is not responsible for the protective function of ApoE2 and ApoE3. Both ApoE3 and ApoE4 have been shown to protect rat neurons from apoptosis via reducing GSK3β activity [23]. Inconsistently, we did not observe the neuroprotective effect of ApoE4 or altered GSK3β activity by ApoE. The reasons could be: (1) we used neurons generated from human iPSC, while primary neuronal culture from rats was utilized in other study; (2) we induced neuronal apoptosis via adding H_2_O_2_ to the medium while trophic additives in rat neuronal medium were removed in other study; (3) human *APOE* plasmids were transfected to HEK293 to yield human ApoE in our study, whereas others employed ApoE secreted from rat glia. We suppose all these differences may lead to the discrepancy.

Activation of FoxO3a in the nucleus is sufficient to elevate its downstream target gene levels, such as *BIM* [44,45]. Bim protein triggers the activation of other pro-apoptotic factors, ultimately resulting in the activation of the well-known apoptosis executioner caspase 3 [46]. Increased Bim level is associated with neuronal loss in AD brains [15,16]. FoxO3a is activated after translocation to the nucleus in response to Aβ and leads to neuron death through Bim upregulation [47]. In our study, Bim expression level was upregulated by H_2_O_2_, which agrees with the dramatically increased cell apoptosis and nuclear FoxO3a translocation. In addition to FoxO3a transcriptional activity, ApoE2 and ApoE3 also attenuated the H_2_O_2_-induced increase in Bim level, demonstrating that FoxO3a/Bim is regulated by ApoE2 and ApoE3. *FASL*, *PUMA* and *MnSOD* also act as pivotal downstream factors of FoxO3a. However, their expression levels were not altered by ApoE isoforms, implying that none of them contributed to the neuroprotection of ApoE2 and ApoE3. It should be noted that FoxO3a responses to oxidative stress is dual. Short-term activation results in the upregulation of protective mechanism. However, failure to cope with oxidative stress and sustained FoxO3a activation may render a cell prone to apoptosis rather than to exert an antioxidant function [42]. Although we cannot exclude other downstream factors of FoxO3a which could also be involved in the neuroprotection of ApoE2 and ApoE3, our results agree and extend previous perspective on FoxO3a’s role in neuronal apoptosis and provide the novel finding that ApoE2 and ApoE3 protect neurons from apoptosis through inactivating FoxO3a/Bim signaling pathway. 

Previous studies have shown the single amino acid polymorphisms of ApoE isoforms substantially alter the structure, thereby modulating its binding properties with regard to both lipids and receptors [48,49,50]. The different binding preference and interactions with members of the LDL receptor family have been proposed to account for the differential performance of ApoE isoforms in many aspects, including lipid transport, synaptic integrity and plasticity, Aβ metabolism [51]. Thus, we speculate that the observed isoform-specific effects of ApoE in the present study may also owe to the differential binding affinities/preference of ApoE isoforms to various lipids/receptors which could induce different signaling pathways in the downstream. The underlying mechanism needs further investigations.

Taken together, we investigated the effects of exogenous ApoE isoforms on H_2_O_2_-induced apoptosis with human iPSC-derived cortical neurons and demonstrated for the first time that ApoE2 and ApoE3, but not ApoE4, afford protection from apoptosis through activating Akt and inhibiting FoxO3a/Bim signaling pathway. Our findings provide new insights into the understanding of higher AD risk in *APOE4* carriers and indicate ApoE as a new therapeutic target to reduce neuronal apoptosis. In addition to AD, our results imply that ApoE may also play isoform-specific roles in other neurodegenerative diseases involving ROS-induced apoptosis.

## 4. Materials and Methods

### 4.1. Apolipoprotein E Preparation

Recombinant ApoE2, ApoE3 and ApoE4 were generated in HEK293 cells following Huang’s protocol [21]. Briefly, 293-F cells (FreeStyle 293-F cell, Thermo Fisher Scientific Waltham, MA, USA), were cultured in suspension in serum-free FreeStyle 293 Expression Medium (Thermo Fisher Scientific) and transfected with control plasmid (FUGW, Addgene plasmid # 14883) or human ApoE expression plasmids (a kind gift from Prof. Thomas Südhof, Stanford University, CA, USA) using lipid-based FreeStyle MAX Reagent (Thermo Fisher Scientific) following the manufacturer’s instructions. Supernatants from transfected 293-F cells were harvested 6 days after transfection. After centrifugation for 4 h at 50,000 g, more than 90% of the ApoE remained in the supernatant with apparently equal amounts of ApoE2, ApoE3, and ApoE4. ApoE proteins were quantified on sodium dodecyl sulphate-polyacrylamide gel electrophoresis (SDS-PAGE) gels using serum bovine albumin as a standard. The concentrations of HEK293-derived ApoE proteins in the harvested medium are typically 400–600 μg/mL. The ApoE supernatants were diluted to proper concentration by fresh BrainPhys medium (Stemcell Technologies, Vancouver, Canada) and added into cultured neurons for ApoE stimulation. The supernatant from control- (FUGW-) transfected 293-F cells was diluted the same way (control medium) and added to neurons as the control condition to exclude the possible influence from other HEK293-secreted factors. Unless otherwise stated, physiological concentration of ApoE (5 μg/mL) was used [3].

### 4.2. Antibodies

The antibodies used for iPSC characterization were OCT4 (Thermo Fisher Scientific, rabbit 1:200), SSEA4 (Thermo Fisher Scientific, mouse IgG3, 1:200) and TRA1-81 (Thermo Fisher Scientific, mouse IgM 1:200). The antibodies used for NPC and neuron characterizations were PAX6 (Millipore, Burlington, MA, USA, rabbit 1:1000), Nestin (Millipore, mouse 1:2000), β3-tubulin (Millipore, mouse 1:2000), MAP2 (Abcam, Cambridge, UK, chicken 1:4000), NeuN (Abcam, rabbit 1:1000), Tbr1 (Abcam, rabbit 1:1000) and vGLUT1 (Synaptic Systems, Göttingen, Germany, rabbit 1:200). ApoE (rabbit 1:1000) was from Abcam. Antibodies from Cell Signaling Technology (Danvers, MA, USA) included: cleaved caspase 3, p-FoxO3a, FoxO3, PCNA, Bim, p-Akt, Akt, p-JNK1/2, JNK1/2; p-GSK3β (Ser9) and GSK3β; 1:1000 for Western blot analysis and 1:400 for immunofluorescence. Secondary antibodies were from Invitrogen (Waltham, MA, USA, 1:2000): anti-rabbit Alexa Fluor 546, anti-mouse IgG3 Alexa Fluor 488, anti-mouse IgM Alexa Fluor 647, anti-rabbit Alexa Fluor 488, anti-chicken Alexa Fluor 647 and anti-mouse Alexa Fluor 568.

### 4.3. iPSC Culture and In Vitro Differentiation of Embryoid Bodies (EBs)

Four different human iPSC lines were used in this study, and they were all cultured on Geltrex-coated plates in complete Essential-8 medium (Life Technologies, Waltham, MA, USA) according to the manufacturer’s instructions. Cells were passaged weekly with versene solution (Thermo Fisher Scientific). All cell cultures were kept in a humidified atmosphere at 5% CO_2_ and 37 °C. Mycoplasma tests were negative for all cultures. 

iPSC colonies were detached with accutase (Thermo Fisher Scientific) and grown as EBs in suspension for 8 days in E8 medium followed by adhering to Geltrex-coated plates for another 8 days of differentiation. Cells migrating out of the attached EBs were collected for analysis of three germ layers markers.

### 4.4. Generation of Cortical Neurons from Human Induced Pluripotent Stem Cells (iPSCs)

Human iPSCs were differentiated into cortical neurons using an established protocol [52] with a few modifications. Briefly, iPSCs were passaged and plated to 6-well Nunc plates (Thermo Fisher Scientific) with accutase in the presence of 10 μM ROCK inhibitor (Sigma-Aldrich, MO, USA), neural induction was initiated on the next day (cell culture reached 95% confluency) by changing to neural induction media (NIM,a 1:1 mixture of N2 and B27 medium supplemented with 250 nM LDN193189, 10µM SB431542 and 5µM XAV939 (all from Tocris, Bristol, UK); N2 medium consisted of DMEM/F12, 1× N2 supplement, 50 μM 2-mercaptoethanol, 100 μM non-essential amino acids, 10 μg/mL insulin; B27 medium consisted of Neurobasal medium, 1× B27 supplement, 200 mM L-glutamine (all from Life Technologies)). Cells were maintained in NIM for 7 days until a uniform neuroepithelial sheet occurred (day 0–6). The neuroepithelial sheet was then dissociated with Accutase and passaged at day 7, 12, 15 and 18 to expand neural progenitor cells (NPCs) and maintained in neural maintenance media (a 1:1 mixture of N2 and B27 medium supplemented with 200 μM L-Ascorbic acid 2-phosphate (AA2P, Sigma-Aldrich)). Cells were replated on day 20 onto poly-L-ornithine (0.01%, Sigma-Aldrich) and laminin-coated (1 μg/cm^2^, Sigma-Aldrich) plates with the density of 8 × 10^5^ in 6-well plates. Neurons were cultured in neuronal maturation medium (B27 medium supplemented with 200 μM AA2P and compounds from PeproTech (Cranbury, NJ, USA): 10 ng/mL BDNF, 10 ng/mL GDNF, 10 μM DAPT) for 7 days. Cultured medium was then switched to BrainPhys medium (Stemcell Technologies) for further maturation till day 60. Four batches of neurons for each iPSC line were generated and analyzed.

### 4.5. Electrophysiology

Whole-cell patch-clamp recordings were performed at day 60 following the initiation of neural induction. Borosilicate glass electrodes (resistance 5–15 mΩ) were filled with internal solution containing 135 mM potassium gluconate, 7 mM NaCl, 10 mM HEPES, 2 mM Na2ATP, 0.3 mM Na2GTP and 2 mM MgCl2 (pH 7.4). Recordings were made at room temperature using a MultiClamp 700B amplifier (Molecular Devices, San Jose, CA, USA). Cells were continuously perfused with oxygenated artificial cerebrospinal fluid (aCSF) composed of 125 mM NaCl, 25 mM NaHCO_3_, 1.25 mM NaH_2_PO_4_, 3 mM KCl, 2 mM CaCl_2_, 1 mM MgCl_2_, 25 mM glucose and 3 mM pyruvic acid, bubbled with 95% O_2_ and 5% CO_2_. A check for action potential (AP) firing in current clamp mode was done by performing stepwise current injections, 8–10 neurons tested per coverslip.

### 4.6. Cell Apoptosis Measurement

After exposing to H_2_O_2_, cell apoptosis assay was carried out with HT TiterTACS Apoptosis Detection Kit (R&D 4822-96-K, Minneapolis, MN, USA) according to the manufacturer’s protocol. Measure absorbance at 450 nm using microplate reader (BioTek, Winooski, VT, USA) to determine cell apoptotic unit.

### 4.7. Immunofluorescence (IF)

Cells were fixed and permeabilized with Image-iT^®^ Fixation/Permeabilization Kit (Thermo Fisher Scientific) and were blocked in blocking buffer (0.1 M glycine, 2% BSA, 0.1% Triton-X100 in PBS). Primary antibodies were diluted in blocking buffer and incubated at 4 °C overnight. After washing, samples were incubated with alexa488-, alexa568- and alexa647-conjugated secondary antibodies for 2 h at room temperature and thereafter mounted using Dako mounting media. Samples were captured using an SP8 confocal system (Leica, Wetzlar, Germany) and image analysis was performed using Image J (NIH, USA)

### 4.8. Quantitative Real Time PCR

Total RNA was isolated from samples using Trizol reagent (Life Technologies) and purification was performed following the manufacturers’ instructions. Reverse transcription was performed on 2 μg of RNA using Superscript reverse transcriptase III and random hexamer primers. TaqMan Universal PCR Master Mix was employed, and qPCR reactions were carried out on Micro-Amp 96-well optical microtiter plates with 7900 HT Fast QPCR System (Applied Biosystems). cDNA (2.5 ng) was used in the PCR and all samples were run in duplicate. The comparative threshold cycle values were normalized for the HPRT1 reference gene and the results were expressed as CT relative quantification using the ΔΔCT method. TaqMan gene expression assays for the following genes were carried out: *NANOG (Hs02387400_g1); POU class 5 homeobox 1 (POU5F1/OCT4, Hs04260367_gH); paired box 6 (PAX6, Hs01088114_m1); LIN28 (Hs00702808_s1); alpha-fetoprotein (AFP, Hs01040598_m1); smooth muscle actin (SMA, Hs00426835_g1); β3-tubulin (TUJ1, Hs00801390_s1); SOX1 (Hs01057642_s1); microtubule associated protein 2 (MAP2, Hs00258900_m1); BCL2L11 (BIM, Hs00708019_s1); factor related apoptosis ligand (FASL, Hs00181226_g1); p53 upregulated modulator of apoptosis (PUMA, Hs00248075_m1); SOD2 (MnSOD, Hs00167309_m1); SYP (Hs00300531_m1); hypoxanthine phosphoribosyl transferase 1 (HPRT1, Hs02800695_m1).*

### 4.9. Western Blot Analysis

Cells were collected in phosphate-buffered saline (PBS), and the cell pellet was divided equally into two tubes for whole-cell lysate and cell fractionation. Whole-cell lysates were prepared with 100 μL of lysis buffer (20 mM Tris-HCl, pH 7.5, 150 mM NaCl, 1 mM EDTA, 1% Triton X-100, 0.5% deoxycholate 0.1% SDS) supplemented with MiniComplete protease inhibitor cocktail (Roche, Basel, Switzerland) and phosphatase inhibitor cocktail (Sigma-Aldrich). For cytoplasmic fractionation, 100 μL of cytosol extraction buffer (10 mM Tris-Cl (pH 8.0), 60 mM KCl, 1 mM EDTA, 1 mM DTT, protease and phosphatase inhibitors) was added for 10 min before the addition of 0.5% NP-40 was applied for another 10 min. After centrifugation at 5000 rpm for 10 min at 4 °C, the supernatant was collected into a new tube as the cytoplasmic fraction; and the pellets were lysed with 100 μL of nuclear extraction buffer (20 mM Tris-Cl (pH 8.0), 0.5% NP-40, 0.4 M NaCl, 1.5 mM MgCl2, 1.5 mM EDTA, 1 mM DTT, protease and phosphatase inhibitors). After 10min on ice incubation, lysates were centrifugated at 15,000 rpm for 10 min at 4 °C. The supernatant was collected as the nuclear extract. Whole-cell lysates, cytoplasmic extracts, and nuclear extracts were stored at -80 °C until further analysis. Protein concentration was measured with a BCA protein assay kit according to the manufacturer’s instructions (BioRad, Hercules, CA, USA). Samples with equal amounts of proteins were separated on 4–12% Bis-tris gel or 10–20% Tricine gel and transferred using Trans-Blot Turbo Transfer System (BioRad). β3-tubulin was used as the loading control for whole-cell and cytoplasmic samples. Membranes were blocked in 5% non-fat dry milk for 1 h at room temperature and incubated over night at 4 °C with primary antibodies. After washing, membranes were incubated with HRP-conjugated anti-mouse/rabbit secondary antibodies for 1 h at room temperature. For protein detection, Pierce™ ECL plus Western blot Substrate (Thermo Fisher Scientific) was used. Membranes were re-incubated with other primary antibodies after stripping with Restore plus stripping buffer (Thermo Fisher Scientific). Band intensities were calculated using Image J.

### 4.10. Statistics

All data were expressed as the mean ± SEM unless otherwise indicated. Un-paired Student’s t test was used to compare differences between two groups, assuming the data were normally distributed. One-way ANOVA with a post hoc Dunnett test was used to analyze differences between more than two groups, with * *p* < 0.05 considered statistically significant. All statistical analyses were performed using GraphPad Prism (Version 8, San Diego, CA, USA).

## Figures and Tables

**Table 1:** List of iPSC lines.

iPSC Line	Gender	Age at Biopsy	*APOE* Genotype	Vendor	Diagnosis
YK26	Female	Adult	*APOE3/E3*	UCONN HEALTH	Non-dementia
YK27	Female	Adult	*APOE3/E3*	UCONN HEALTH	Non-dementia
TZ1	Female	Fetal	*APOE3/E3*	UCONN HEALTH	Non-dementia
BONi037-A (B037)	Female	75–79	*APOE3/E3*	EBiSC	Non-dementia

## Supplementary Materials

The following are available online at https://www.mdpi.com/article/10.3390/ijms222111582/s1.
