# Supplementary material for: Isoform-Specific Effects of Apolipoprotein E on Hydrogen Peroxide-Induced Apoptosis in Human Induced Pluripotent Stem Cell (iPSC)-Derived Cortical Neurons
Source: Int J Mol Sci. 2021 Oct 27;22(21):11582. doi: 10.3390/ijms222111582 (PMC8584079; doi:10.3390/ijms222111582)
Supplement: Supplementary file 1 [file ijms-22-11582-s001.zip › ijms-1414542-supplementary.pdf]

Supplementary Figure S1

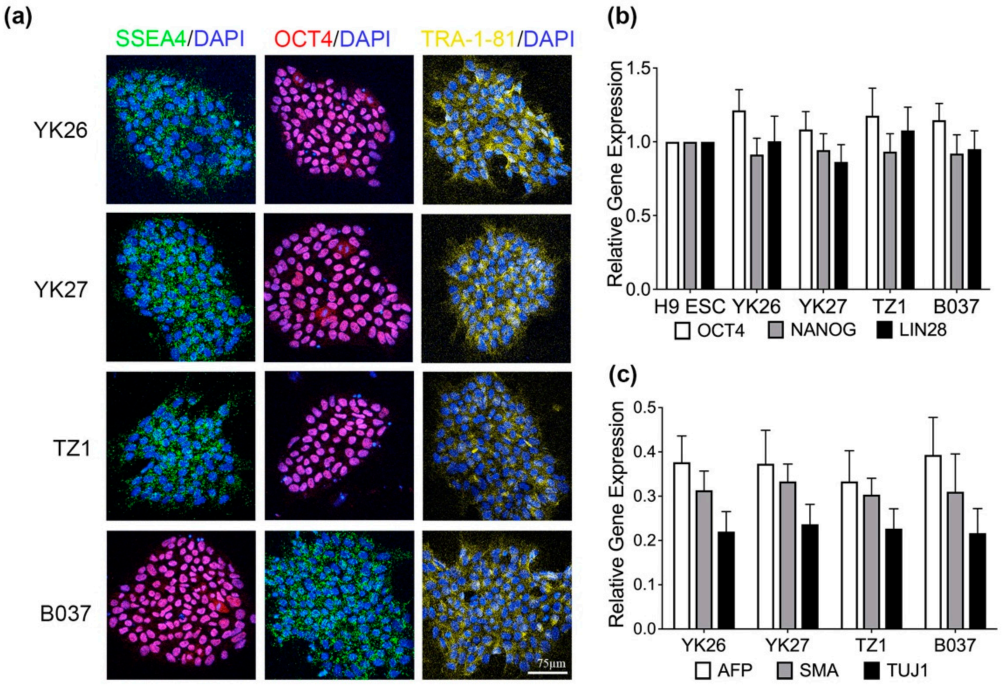

Supplementary Figure S1. Characterization of human iPSC lines. Four human iPSC lines were employed in the study. (a) Immunofluorescence images for human iPSCs confirmed their pluripotency with positive staining for stem cell markers: SSEA4, OCT4 and TRA-1-81. White scale bar, 75 µm. (b) Expression of pluripotency genes *OCT4*, *NANOG* and *LIN28* compared to the human H9-ESC line. (c) Transcript levels of endogenous germ layer markers in EB differentiation of all four iPSC lines. *TUJ1* (ectoderm), *SMA* (mesoderm) and *AFP* (endoderm). n = 3 experiments for each line were repeated independently to check the quality of human iPSC lines. Data are presented as mean ± standard error of mean (SEM).

Supplementary Figure S2

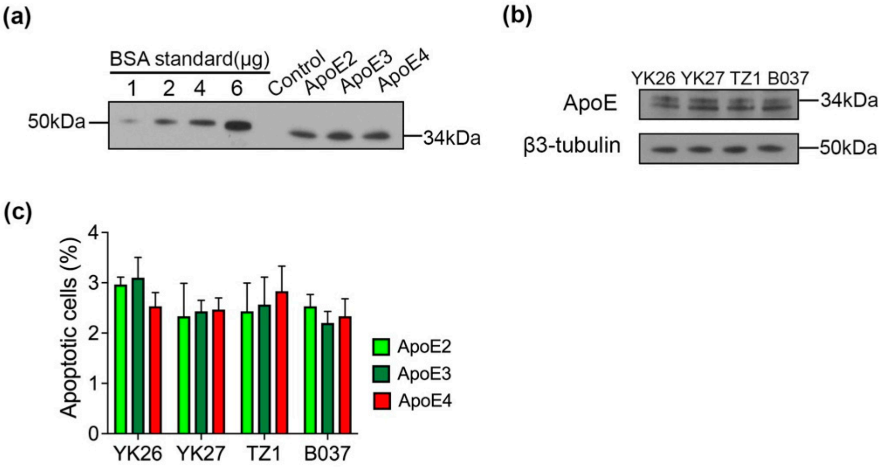

Supplementary Figure S2. ApoE isoforms generated in HEK293 do not cause neuronal apoptosis. (a) Production of recombinant ApoE2, ApoE3, and ApoE4 in transfected HEK293 system. ApoE proteins were quantified on SDS-Page gels using serum bovine albumin (BSA) as a standard to calculate ApoE concentrations. The concentrations of HEK293 ApoE in the harvested medium were typically 400-600 μg/ml. The control medium was harvested from transfected HEK293-F cells expressing GFP and used in the experiments at the same dilution. (b) Western analysis of the ApoE expression in neurons derived from all hiPSC lines on day 60. (c) Neuron apoptosis level was measured after 5 μg/ml of ApoE treatment for 12 h. n=3, data are presented as mean ± SEM.

Supplementary Figure S3

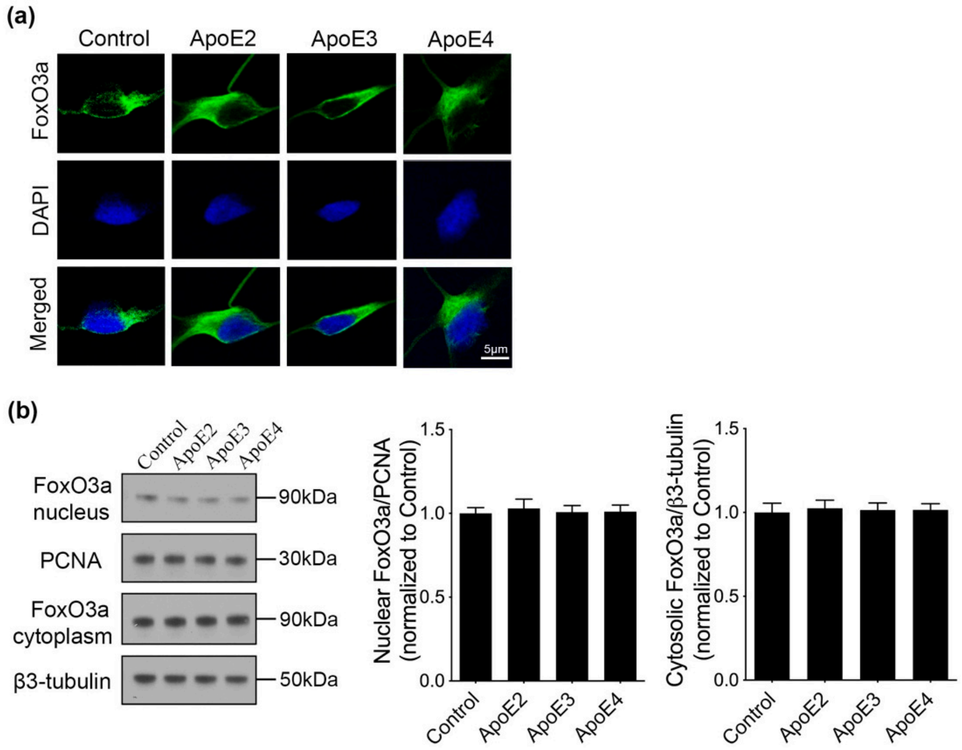

Supplementary Figure S3. ApoE isoforms do not induce FoxO3a translocation in the absence of H_2O_2. (a) Neurons from control or ApoE treated groups (5 μg/ml, 12 h) were immunostained for FoxO3a (green). Nuclei were stained with DAPI (blue). Localization of FoxO3a was visualized by using fluorescence microscopy. Representative images for all lines are shown here. White scale bar, 5 μm. (b) Western blot analysis of nuclear and cytosolic FoxO3a. n = 4 per line, data are presented as a summary of all lines (mean ± SEM) using one-way ANOVA and Dunnett test.

Supplementary Figure S4

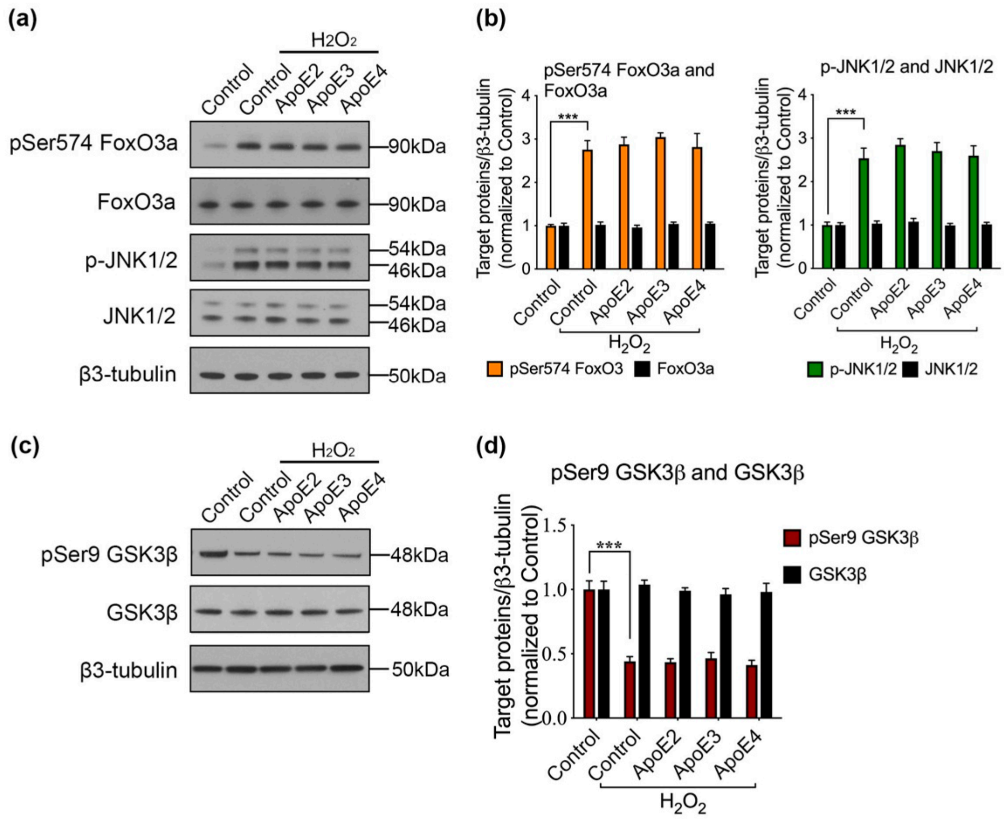

Supplementary Figure S4. Neither JNK/FoxO3a signaling pathway nor GSK3β is involved in the anti-apoptotic effect of ApoE2 and ApoE3. (a) Phosphorylation levels of FoxO3a (pSer574) and JNK (p-JNK1/2) were examined by Western blot. (b) Quantitative analysis of the changes in the expression levels of pSer574 FoxO3a, FoxO3a, p-JNK1/2 and JNK1/2. (c,d) Western analysis of GSK3β activity. n = 4, data are presented as a summary of all lines (mean ± SEM) using one-way ANOVA and Dunnett test. ***$p < 0.001$ versus neurons co-treated with control medium and H_2O_2.
